# Methyl 2-[(3,5-di-*tert*-butyl-4-hy­droxy­benz­yl)sulfan­yl]pyridine-3-carboxyl­ate *n*-hexane hemisolvate

**DOI:** 10.1107/S1600536812018739

**Published:** 2012-05-02

**Authors:** Azhar Ariffin, Nordiana Nordin, Wagee A. Yehye, Seik Weng Ng

**Affiliations:** a Department of Chemistry, University of Malaya, 50603 Kuala Lumpur, Malaysia; b Chemistry Department, Faculty of Science, King Abdulaziz University, PO Box 80203 Jeddah, Saudi Arabia

## Abstract

The title solvated ester, C_22_H_29_NO_3_S·0.5C_6_H_14_, crystallizes with two independent mol­ecules along with a hexane mol­ecule in the asymmetric unit. The two aromatic rings are separated by an –CH_2_–S– linkage; the rings are aligned at 83.27 (4)° in one mol­ecule and 47.66 (7)° in the other. The hy­droxy group of one mol­ecule forms an O—H⋯O hydrogen bond to the other mol­ecule.

## Related literature

For the synthesis of carb­oxy­lic acid, see: Mansor *et al.* (2008 ▶).
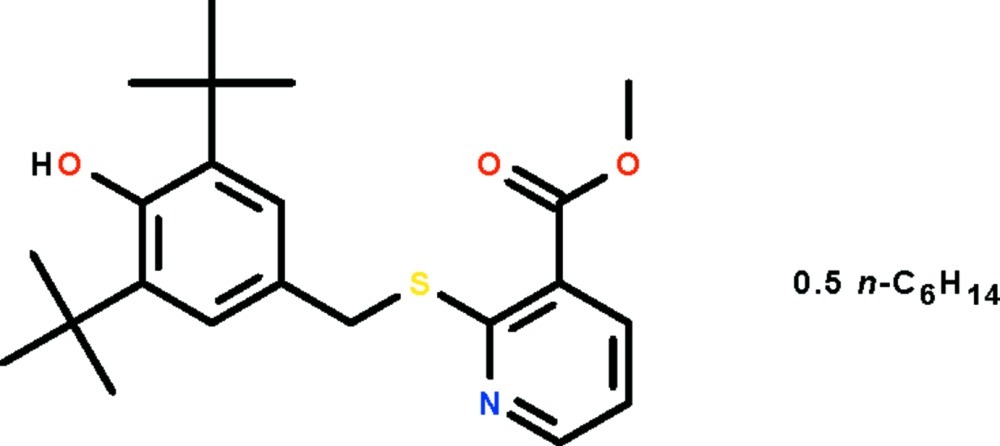


## Experimental

### 

#### Crystal data

C_22_H_29_NO_3_S·0.5C_6_H_14_
*M* *_r_* = 430.61
Monoclinic, 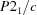

*a* = 15.0665 (5) Å
*b* = 9.4818 (3) Å
*c* = 34.6700 (13) Å
β = 90.796 (3)°
*V* = 4952.4 (3) Å^3^
*Z* = 8
Mo *K*α radiation
μ = 0.16 mm^−1^
*T* = 100 K
0.40 × 0.35 × 0.30 mm

#### Data collection

Agilent SuperNova Dual diffractometer with an Atlas detector
Absorption correction: multi-scan (*CrysAlis PRO*; Agilent, 2011 ▶) *T*_min_ = 0.941, *T*_max_ = 0.955
34553 measured reflections
11425 independent reflections
8907 reflections with *I* > 2σ(*I*)
*R*_int_ = 0.041

#### Refinement

*R*[*F* ^2^ > 2σ(*F* ^2^)] = 0.047
*wR*(*F* ^2^) = 0.113
*S* = 1.00
11426 reflections
551 parameters
2 restraints
H atoms treated by a mixture of independent and constrained refinement
Δρ_max_ = 0.33 e Å^−3^
Δρ_min_ = −0.30 e Å^−3^

### 

Data collection: *CrysAlis PRO* (Agilent, 2011 ▶); cell refinement: *CrysAlis PRO*; data reduction: *CrysAlis PRO*; program(s) used to solve structure: *SHELXS97* (Sheldrick, 2008 ▶); program(s) used to refine structure: *SHELXL97* (Sheldrick, 2008 ▶); molecular graphics: *X-SEED* (Barbour, 2001 ▶); software used to prepare material for publication: *publCIF* (Westrip, 2010 ▶).

## Supplementary Material

Crystal structure: contains datablock(s) global, I. DOI: 10.1107/S1600536812018739/bt5894sup1.cif

Structure factors: contains datablock(s) I. DOI: 10.1107/S1600536812018739/bt5894Isup2.hkl

Supplementary material file. DOI: 10.1107/S1600536812018739/bt5894Isup3.cml

Additional supplementary materials:  crystallographic information; 3D view; checkCIF report

## Figures and Tables

**Table 1:** Hydrogen-bond geometry (Å, °) *Cg* is the centroid of the C1–C6 ring.

*D*—H⋯*A*	*D*—H	H⋯*A*	*D*⋯*A*	*D*—H⋯*A*
O1—H1⋯O6	0.83 (1)	2.22 (2)	2.767 (2)	124 (2)

Symmetry code: (i) 
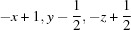
.

**Fig. 1.**
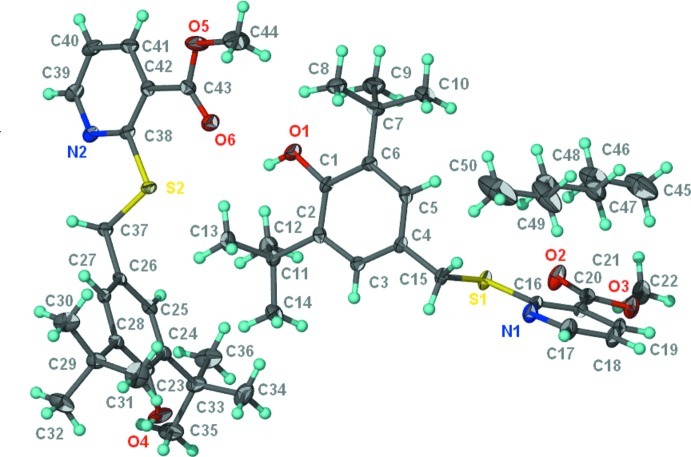
Anisotropic displacement ellipsoid plot (Barbour, 2001) of at the two independent molecules and the solvent molecule at the 70% probability level; hydrogen atoms are drawn as spheres of arbitrary radius.

## supplementary crystallographic information

### Comment

The study continues from an earlier study on 2-([(3,5–3,5-di-*tert*-butyl-4-hydroxphenyl)methyl]sulfanyl)pyridine-3-carboxylic acid Mansor *et al.*, 2008). The compound is readily esterified with methanol medium when the reaction is catalyzed with *p*-toulenesulfonic acid. The ester crystallizes with a hexane molecule of crystallization (Scheme I, Fig. 1). The two aromatic rings that are separated by an –CH_2_–S– linkage are aligned at 83.27 (4) ° in one molecule and 47.66 (7) ° in the other. The hydroxyl group of one molecule forms a O—H···O hydrogen bond, whereas the other one shows a O—H···π contact (Table 1).

### Experimental

2-((3,5-Di-*tert*-butyl-4-hydroxybenzyl)thio)nicotinic acid (1.36 g m, 3.97 mmol,) was dissolved in methanol (50 ml). A small quantity of *p*-toluenesulfonic acid was added as catalyst. The solution was heated for 6 h. The solvent was removed and sodium bicarbonate in water was added until the solution was approximately netural. The organic compound was extracted by ethyl acetate; the organic phase was dried with magnesium sulfate. The ethyl acetate was evaporated and the product recrystallized from *n*-hexane to give light yellow crystals. The methy ester crystallizes as two independent molecules along with a solvent (Scheme I, Fig. 1). The two aromatic rings that are separated by the –CH_2_–S– linkage are aligned at 83.27 (4) ° in one molecule and 47.66 (7) ° in the other. The molecule having the larger twist uses its hydroxy H atom to form a hydrogen bond to the carbonyl O atom of the molecule having the smaller twist (Table 1).

### Refinement

Carbon-bound H-atoms were placed in calculated positions [C–H 0.95 to 0.99 Å, *U*_iso_(H) 1.2 to 1.5*U*_eq_(C)] and were included in the refinement in the riding model approximation.

The hydroxy H-atoms were located in a difference Fourier map, and were refined isotropically with a distance restraint of O–H 0.84±0.01 Å.

### Crystal data

**Table:**

C_22_H_29_NO_3_S·0.5C_6_H_14_	*F*(000) = 1864
*M**_r_* = 430.61	*D*_x_ = 1.155 Mg m^−^^3^
Monoclinic, *P*2_1_/*c*	Mo *K*α radiation, λ = 0.71073 Å
Hall symbol: -P 2ybc	Cell parameters from 9294 reflections
*a* = 15.0665 (5) Å	θ = 2.4–27.5°
*b* = 9.4818 (3) Å	µ = 0.16 mm^−^^1^
*c* = 34.6700 (13) Å	*T* = 100 K
β = 90.796 (3)°	Prism, light yellow
*V* = 4952.4 (3) Å^3^	0.40 × 0.35 × 0.30 mm
*Z* = 8	

### Data collection

**Table:**

Agilent SuperNova Dual diffractometer with an Atlas detector	11425 independent reflections
Radiation source: SuperNova (Mo) X-ray Source	8907 reflections with *I* > 2σ(*I*)
Mirror monochromator	*R*_int_ = 0.041
Detector resolution: 10.4041 pixels mm^-1^	θ_max_ = 27.6°, θ_min_ = 2.4°
ω scan	*h* = −19→16
Absorption correction: multi-scan (*CrysAlis PRO*; Agilent, 2011)	*k* = −12→11
*T*_min_ = 0.941, *T*_max_ = 0.955	*l* = −45→41
34553 measured reflections	

### Refinement

**Table:**

Refinement on *F*^2^	Primary atom site location: structure-invariant direct methods
Least-squares matrix: full	Secondary atom site location: difference Fourier map
*R*[*F*^2^ > 2σ(*F*^2^)] = 0.047	Hydrogen site location: inferred from neighbouring sites
*wR*(*F*^2^) = 0.113	H atoms treated by a mixture of independent and constrained refinement
*S* = 1.00	*w* = 1/[σ^2^(*F*_o_^2^) + (0.0395*P*)^2^ + 3.1619*P*] where *P* = (*F*_o_^2^ + 2*F*_c_^2^)/3
11426 reflections	(Δ/σ)_max_ = 0.001
551 parameters	Δρ_max_ = 0.33 e Å^−^^3^
2 restraints	Δρ_min_ = −0.30 e Å^−^^3^

### Fractional atomic coordinates and isotropic or equivalent isotropic displacement parameters (Å^2^)

**Table:**

	*x*	*y*	*z*	*U*_iso_*/*U*_eq_	
S1	0.72068 (3)	0.35500 (4)	0.438613 (12)	0.01646 (10)	
O1	0.82843 (9)	0.76073 (14)	0.29857 (4)	0.0244 (3)	
H1	0.8214 (15)	0.728 (2)	0.2764 (4)	0.036 (7)*	
O2	0.83997 (9)	0.17218 (14)	0.47471 (4)	0.0259 (3)	
O3	0.81798 (8)	0.06381 (13)	0.53138 (4)	0.0206 (3)	
N1	0.59707 (9)	0.39135 (15)	0.49119 (4)	0.0185 (3)	
C1	0.78031 (11)	0.69231 (17)	0.32609 (5)	0.0148 (3)	
C2	0.71649 (10)	0.58834 (17)	0.31702 (5)	0.0134 (3)	
C3	0.67382 (10)	0.52510 (16)	0.34816 (5)	0.0134 (3)	
H3	0.6304	0.4545	0.3431	0.016*	
C4	0.69244 (10)	0.56147 (17)	0.38616 (5)	0.0131 (3)	
C5	0.75521 (10)	0.66599 (17)	0.39378 (5)	0.0141 (3)	
H5	0.7677	0.6913	0.4198	0.017*	
C6	0.80023 (10)	0.73445 (17)	0.36441 (5)	0.0142 (3)	
C7	0.86849 (11)	0.85133 (18)	0.37354 (5)	0.0187 (4)	
C8	0.84068 (12)	0.99148 (18)	0.35410 (6)	0.0226 (4)	
H8A	0.7853	1.0251	0.3654	0.034*	
H8B	0.8317	0.9762	0.3264	0.034*	
H8C	0.8874	1.0620	0.3582	0.034*	
C9	0.96164 (12)	0.8069 (2)	0.35996 (6)	0.0264 (4)	
H9A	0.9811	0.7231	0.3743	0.040*	
H9B	1.0037	0.8841	0.3646	0.040*	
H9C	0.9592	0.7853	0.3323	0.040*	
C10	0.87606 (13)	0.8805 (2)	0.41700 (6)	0.0265 (4)	
H10A	0.8959	0.7948	0.4304	0.040*	
H10B	0.8180	0.9089	0.4267	0.040*	
H10C	0.9191	0.9563	0.4216	0.040*	
C11	0.69576 (11)	0.54066 (18)	0.27526 (5)	0.0168 (3)	
C12	0.77809 (12)	0.46918 (19)	0.25784 (5)	0.0229 (4)	
H12A	0.7953	0.3880	0.2737	0.034*	
H12B	0.8272	0.5368	0.2572	0.034*	
H12C	0.7640	0.4374	0.2316	0.034*	
C13	0.66440 (13)	0.66520 (19)	0.24972 (5)	0.0241 (4)	
H13A	0.6111	0.7074	0.2608	0.036*	
H13B	0.6507	0.6309	0.2236	0.036*	
H13C	0.7115	0.7363	0.2486	0.036*	
C14	0.62038 (12)	0.43170 (19)	0.27365 (5)	0.0232 (4)	
H14A	0.5667	0.4726	0.2848	0.035*	
H14B	0.6379	0.3478	0.2884	0.035*	
H14C	0.6084	0.4052	0.2468	0.035*	
C15	0.64611 (11)	0.49028 (17)	0.41924 (5)	0.0149 (3)	
H15A	0.5899	0.4468	0.4101	0.018*	
H15B	0.6321	0.5601	0.4395	0.018*	
C16	0.67199 (11)	0.32072 (17)	0.48357 (5)	0.0148 (3)	
C17	0.55904 (11)	0.3716 (2)	0.52556 (5)	0.0221 (4)	
H17	0.5066	0.4231	0.5309	0.027*	
C18	0.59154 (12)	0.2812 (2)	0.55354 (5)	0.0222 (4)	
H18	0.5622	0.2703	0.5774	0.027*	
C19	0.66795 (11)	0.20683 (19)	0.54570 (5)	0.0187 (4)	
H19	0.6915	0.1424	0.5642	0.022*	
C20	0.71060 (11)	0.22649 (17)	0.51058 (5)	0.0149 (3)	
C21	0.79532 (11)	0.15355 (17)	0.50291 (5)	0.0168 (3)	
C22	0.90344 (12)	−0.0032 (2)	0.52704 (6)	0.0245 (4)	
H22A	0.9144	−0.0673	0.5488	0.037*	
H22B	0.9500	0.0690	0.5266	0.037*	
H22C	0.9039	−0.0566	0.5029	0.037*	
S2	0.84000 (3)	0.64682 (4)	0.164523 (12)	0.01465 (10)	
O4	0.50710 (8)	0.22359 (13)	0.16869 (4)	0.0251 (3)	
H4	0.4579 (9)	0.260 (2)	0.1644 (7)	0.045 (7)*	
O5	0.98462 (9)	0.99237 (14)	0.22179 (4)	0.0313 (3)	
O6	0.89539 (8)	0.80484 (13)	0.22566 (4)	0.0220 (3)	
N2	0.95527 (9)	0.70873 (14)	0.10963 (4)	0.0157 (3)	
C23	0.57637 (11)	0.30871 (17)	0.15767 (5)	0.0154 (3)	
C24	0.66138 (11)	0.24573 (17)	0.15966 (5)	0.0157 (3)	
C25	0.73273 (11)	0.32794 (17)	0.14796 (5)	0.0150 (3)	
H25	0.7907	0.2885	0.1489	0.018*	
C26	0.72205 (10)	0.46567 (17)	0.13502 (5)	0.0129 (3)	
C27	0.63775 (10)	0.52384 (17)	0.13415 (5)	0.0140 (3)	
H27	0.6308	0.6187	0.1258	0.017*	
C28	0.56262 (11)	0.44839 (17)	0.14504 (5)	0.0142 (3)	
C29	0.46915 (11)	0.51433 (18)	0.14173 (5)	0.0186 (4)	
C30	0.47325 (12)	0.66895 (19)	0.12899 (7)	0.0293 (5)	
H30A	0.5024	0.6752	0.1039	0.044*	
H30B	0.5071	0.7236	0.1481	0.044*	
H30C	0.4129	0.7069	0.1267	0.044*	
C31	0.42186 (13)	0.5140 (2)	0.18096 (6)	0.0312 (5)	
H31A	0.3649	0.5632	0.1784	0.047*	
H31B	0.4591	0.5622	0.2002	0.047*	
H31C	0.4116	0.4165	0.1891	0.047*	
C32	0.41416 (12)	0.4369 (2)	0.11052 (6)	0.0282 (4)	
H32A	0.4460	0.4391	0.0861	0.042*	
H32B	0.3565	0.4837	0.1072	0.042*	
H32C	0.4050	0.3388	0.1184	0.042*	
C33	0.67511 (12)	0.09333 (17)	0.17365 (6)	0.0209 (4)	
C34	0.64672 (15)	0.0793 (2)	0.21592 (6)	0.0327 (5)	
H34A	0.6838	0.1407	0.2322	0.049*	
H34B	0.6539	−0.0188	0.2243	0.049*	
H34C	0.5844	0.1071	0.2182	0.049*	
C35	0.62306 (13)	−0.01154 (19)	0.14800 (6)	0.0292 (5)	
H35A	0.6364	−0.1081	0.1563	0.044*	
H35B	0.6404	0.0007	0.1211	0.044*	
H35C	0.5593	0.0061	0.1504	0.044*	
C36	0.77303 (13)	0.0501 (2)	0.17171 (7)	0.0328 (5)	
H36A	0.8087	0.1124	0.1883	0.049*	
H36B	0.7932	0.0576	0.1450	0.049*	
H36C	0.7797	−0.0476	0.1806	0.049*	
C37	0.80198 (11)	0.54897 (17)	0.12235 (5)	0.0156 (3)	
H37A	0.7855	0.6143	0.1012	0.019*	
H37B	0.8491	0.4849	0.1132	0.019*	
C38	0.93139 (10)	0.74019 (16)	0.14580 (5)	0.0136 (3)	
C39	1.02497 (11)	0.77768 (18)	0.09500 (5)	0.0194 (4)	
H39	1.0426	0.7547	0.0696	0.023*	
C40	1.07252 (11)	0.88001 (19)	0.11471 (6)	0.0213 (4)	
H40	1.1213	0.9263	0.1031	0.026*	
C41	1.04732 (11)	0.91313 (18)	0.15174 (5)	0.0191 (4)	
H41	1.0786	0.9833	0.1660	0.023*	
C42	0.97578 (10)	0.84316 (17)	0.16818 (5)	0.0150 (3)	
C43	0.94730 (11)	0.87594 (17)	0.20780 (5)	0.0172 (4)	
C44	0.95887 (17)	1.0294 (3)	0.26088 (6)	0.0443 (6)	
H44A	0.9950	1.1087	0.2701	0.066*	
H44B	0.9682	0.9481	0.2779	0.066*	
H44C	0.8960	1.0561	0.2609	0.066*	
C45	0.90495 (18)	0.4113 (3)	0.56068 (9)	0.0575 (8)	
H45A	0.9585	0.3687	0.5499	0.086*	
H45B	0.8611	0.3375	0.5657	0.086*	
H45C	0.9202	0.4596	0.5849	0.086*	
C46	0.86628 (15)	0.5170 (2)	0.53206 (8)	0.0415 (6)	
H46A	0.9113	0.5904	0.5268	0.050*	
H46B	0.8527	0.4680	0.5075	0.050*	
C47	0.78269 (14)	0.5873 (2)	0.54639 (6)	0.0331 (5)	
H47A	0.7971	0.6402	0.5703	0.040*	
H47B	0.7390	0.5135	0.5530	0.040*	
C48	0.74063 (15)	0.6876 (2)	0.51729 (7)	0.0344 (5)	
H48A	0.7845	0.7615	0.5109	0.041*	
H48B	0.7273	0.6345	0.4933	0.041*	
C49	0.65646 (18)	0.7586 (3)	0.53037 (8)	0.0523 (7)	
H49A	0.6696	0.8135	0.5541	0.063*	
H49B	0.6124	0.6852	0.5370	0.063*	
C50	0.6163 (2)	0.8559 (3)	0.50025 (10)	0.0761 (11)	
H50A	0.5629	0.9004	0.5105	0.114*	
H50B	0.6005	0.8014	0.4771	0.114*	
H50C	0.6595	0.9289	0.4936	0.114*	

### Atomic displacement parameters (Å^2^)

**Table:**

	*U*^11^	*U*^22^	*U*^33^	*U*^12^	*U*^13^	*U*^23^
S1	0.0185 (2)	0.0192 (2)	0.0119 (2)	0.00352 (17)	0.00481 (16)	0.00417 (16)
O1	0.0316 (7)	0.0283 (7)	0.0134 (7)	−0.0131 (6)	0.0065 (6)	0.0007 (6)
O2	0.0283 (7)	0.0288 (7)	0.0210 (7)	0.0090 (6)	0.0107 (6)	0.0077 (6)
O3	0.0176 (6)	0.0261 (7)	0.0181 (7)	0.0058 (5)	0.0027 (5)	0.0067 (5)
N1	0.0160 (7)	0.0247 (8)	0.0150 (8)	0.0017 (6)	0.0025 (6)	0.0043 (6)
C1	0.0161 (8)	0.0151 (8)	0.0133 (8)	0.0010 (7)	0.0033 (6)	0.0033 (6)
C2	0.0143 (8)	0.0138 (8)	0.0120 (8)	0.0025 (6)	−0.0004 (6)	0.0003 (6)
C3	0.0132 (7)	0.0120 (7)	0.0150 (8)	0.0009 (6)	−0.0001 (6)	0.0009 (6)
C4	0.0122 (7)	0.0139 (8)	0.0133 (8)	0.0041 (6)	0.0022 (6)	0.0033 (6)
C5	0.0145 (8)	0.0153 (8)	0.0125 (8)	0.0040 (6)	−0.0013 (6)	−0.0005 (6)
C6	0.0132 (7)	0.0137 (8)	0.0156 (8)	0.0009 (6)	0.0001 (6)	0.0008 (6)
C7	0.0169 (8)	0.0189 (8)	0.0203 (9)	−0.0041 (7)	−0.0024 (7)	−0.0001 (7)
C8	0.0208 (9)	0.0199 (9)	0.0270 (10)	−0.0051 (7)	0.0001 (8)	0.0016 (8)
C9	0.0174 (9)	0.0276 (10)	0.0343 (12)	−0.0043 (8)	−0.0016 (8)	0.0021 (8)
C10	0.0286 (10)	0.0266 (10)	0.0240 (10)	−0.0092 (8)	−0.0056 (8)	−0.0021 (8)
C11	0.0213 (8)	0.0175 (8)	0.0116 (8)	−0.0016 (7)	0.0005 (7)	−0.0005 (6)
C12	0.0284 (10)	0.0236 (9)	0.0168 (9)	−0.0014 (8)	0.0040 (8)	−0.0031 (7)
C13	0.0316 (10)	0.0245 (9)	0.0162 (9)	−0.0008 (8)	−0.0040 (8)	0.0032 (7)
C14	0.0290 (10)	0.0246 (9)	0.0159 (9)	−0.0063 (8)	−0.0024 (8)	−0.0044 (7)
C15	0.0154 (8)	0.0176 (8)	0.0118 (8)	0.0015 (7)	0.0022 (6)	0.0030 (6)
C16	0.0165 (8)	0.0170 (8)	0.0111 (8)	−0.0040 (7)	0.0028 (6)	0.0006 (6)
C17	0.0156 (8)	0.0314 (10)	0.0195 (9)	0.0038 (8)	0.0058 (7)	0.0052 (8)
C18	0.0195 (9)	0.0324 (10)	0.0146 (9)	−0.0002 (8)	0.0051 (7)	0.0067 (8)
C19	0.0190 (8)	0.0240 (9)	0.0130 (9)	−0.0009 (7)	0.0001 (7)	0.0067 (7)
C20	0.0152 (8)	0.0162 (8)	0.0135 (8)	−0.0012 (7)	0.0007 (6)	0.0006 (6)
C21	0.0186 (8)	0.0167 (8)	0.0150 (9)	−0.0012 (7)	0.0003 (7)	0.0004 (7)
C22	0.0189 (9)	0.0319 (10)	0.0229 (10)	0.0086 (8)	0.0023 (7)	0.0047 (8)
S2	0.01407 (19)	0.0163 (2)	0.0137 (2)	−0.00558 (16)	0.00258 (15)	−0.00077 (16)
O4	0.0151 (6)	0.0191 (6)	0.0412 (9)	−0.0031 (5)	0.0048 (6)	0.0072 (6)
O5	0.0383 (8)	0.0320 (7)	0.0236 (8)	−0.0210 (6)	0.0053 (6)	−0.0104 (6)
O6	0.0276 (7)	0.0219 (6)	0.0166 (7)	−0.0074 (5)	0.0033 (5)	−0.0007 (5)
N2	0.0152 (7)	0.0160 (7)	0.0160 (7)	0.0006 (6)	0.0032 (6)	0.0013 (6)
C23	0.0142 (8)	0.0162 (8)	0.0156 (9)	−0.0043 (7)	0.0016 (6)	−0.0012 (7)
C24	0.0181 (8)	0.0133 (8)	0.0155 (9)	−0.0017 (7)	−0.0021 (7)	−0.0016 (6)
C25	0.0139 (8)	0.0165 (8)	0.0146 (8)	0.0003 (7)	−0.0022 (6)	−0.0033 (7)
C26	0.0130 (7)	0.0152 (8)	0.0103 (8)	−0.0036 (6)	−0.0002 (6)	−0.0027 (6)
C27	0.0172 (8)	0.0128 (8)	0.0119 (8)	−0.0012 (6)	−0.0016 (6)	−0.0002 (6)
C28	0.0141 (8)	0.0156 (8)	0.0129 (8)	−0.0011 (6)	−0.0006 (6)	−0.0030 (6)
C29	0.0140 (8)	0.0174 (8)	0.0245 (10)	0.0003 (7)	0.0008 (7)	−0.0009 (7)
C30	0.0168 (9)	0.0207 (9)	0.0503 (14)	0.0036 (8)	−0.0038 (9)	0.0031 (9)
C31	0.0248 (10)	0.0353 (11)	0.0338 (12)	0.0097 (9)	0.0087 (9)	−0.0012 (9)
C32	0.0174 (9)	0.0286 (10)	0.0384 (12)	0.0016 (8)	−0.0074 (8)	−0.0085 (9)
C33	0.0201 (9)	0.0130 (8)	0.0296 (11)	−0.0007 (7)	−0.0011 (8)	0.0032 (7)
C34	0.0455 (12)	0.0218 (10)	0.0308 (12)	−0.0004 (9)	−0.0006 (10)	0.0110 (8)
C35	0.0318 (10)	0.0140 (9)	0.0416 (13)	−0.0016 (8)	−0.0028 (9)	−0.0027 (8)
C36	0.0259 (10)	0.0162 (9)	0.0562 (15)	0.0034 (8)	−0.0051 (10)	0.0060 (9)
C37	0.0157 (8)	0.0173 (8)	0.0140 (8)	−0.0038 (7)	0.0012 (6)	−0.0014 (7)
C38	0.0109 (7)	0.0120 (7)	0.0179 (9)	0.0008 (6)	−0.0002 (6)	0.0030 (6)
C39	0.0153 (8)	0.0210 (9)	0.0219 (10)	0.0011 (7)	0.0075 (7)	0.0025 (7)
C40	0.0151 (8)	0.0220 (9)	0.0271 (10)	−0.0029 (7)	0.0066 (7)	0.0052 (8)
C41	0.0144 (8)	0.0178 (8)	0.0249 (10)	−0.0032 (7)	−0.0024 (7)	0.0028 (7)
C42	0.0135 (8)	0.0151 (8)	0.0162 (9)	0.0008 (7)	−0.0017 (6)	0.0030 (7)
C43	0.0165 (8)	0.0169 (8)	0.0181 (9)	−0.0020 (7)	−0.0036 (7)	0.0008 (7)
C44	0.0615 (15)	0.0463 (13)	0.0254 (12)	−0.0297 (12)	0.0110 (11)	−0.0175 (10)
C45	0.0509 (15)	0.0401 (14)	0.081 (2)	0.0019 (12)	−0.0356 (15)	−0.0080 (14)
C46	0.0361 (12)	0.0357 (12)	0.0522 (16)	−0.0046 (10)	−0.0122 (11)	−0.0077 (11)
C47	0.0400 (12)	0.0284 (10)	0.0306 (12)	−0.0051 (9)	−0.0111 (9)	−0.0040 (9)
C48	0.0422 (12)	0.0262 (10)	0.0343 (12)	−0.0072 (9)	−0.0154 (10)	−0.0012 (9)
C49	0.0567 (15)	0.0454 (14)	0.0541 (17)	0.0146 (12)	−0.0279 (13)	−0.0226 (12)
C50	0.076 (2)	0.0335 (14)	0.117 (3)	0.0065 (14)	−0.062 (2)	−0.0126 (16)

### Geometric parameters (Å, º)

**Table:**

S1—C16	1.7621 (17)	C23—C24	1.414 (2)
S1—C15	1.8271 (17)	C24—C25	1.393 (2)
O1—C1	1.3696 (19)	C24—C33	1.537 (2)
O1—H1	0.832 (9)	C25—C26	1.389 (2)
O2—C21	1.208 (2)	C25—H25	0.9500
O3—C21	1.344 (2)	C26—C27	1.385 (2)
O3—C22	1.445 (2)	C26—C37	1.511 (2)
N1—C16	1.342 (2)	C27—C28	1.396 (2)
N1—C17	1.343 (2)	C27—H27	0.9500
C1—C2	1.410 (2)	C28—C29	1.544 (2)
C1—C6	1.415 (2)	C29—C30	1.533 (2)
C2—C3	1.399 (2)	C29—C32	1.540 (3)
C2—C11	1.545 (2)	C29—C31	1.544 (3)
C3—C4	1.387 (2)	C30—H30A	0.9800
C3—H3	0.9500	C30—H30B	0.9800
C4—C5	1.393 (2)	C30—H30C	0.9800
C4—C15	1.510 (2)	C31—H31A	0.9800
C5—C6	1.392 (2)	C31—H31B	0.9800
C5—H5	0.9500	C31—H31C	0.9800
C6—C7	1.542 (2)	C32—H32A	0.9800
C7—C10	1.535 (3)	C32—H32B	0.9800
C7—C9	1.545 (2)	C32—H32C	0.9800
C7—C8	1.545 (2)	C33—C36	1.534 (3)
C8—H8A	0.9800	C33—C34	1.538 (3)
C8—H8B	0.9800	C33—C35	1.541 (3)
C8—H8C	0.9800	C34—H34A	0.9800
C9—H9A	0.9800	C34—H34B	0.9800
C9—H9B	0.9800	C34—H34C	0.9800
C9—H9C	0.9800	C35—H35A	0.9800
C10—H10A	0.9800	C35—H35B	0.9800
C10—H10B	0.9800	C35—H35C	0.9800
C10—H10C	0.9800	C36—H36A	0.9800
C11—C14	1.536 (2)	C36—H36B	0.9800
C11—C12	1.544 (2)	C36—H36C	0.9800
C11—C13	1.546 (2)	C37—H37A	0.9900
C12—H12A	0.9800	C37—H37B	0.9900
C12—H12B	0.9800	C38—C42	1.410 (2)
C12—H12C	0.9800	C39—C40	1.381 (3)
C13—H13A	0.9800	C39—H39	0.9500
C13—H13B	0.9800	C40—C41	1.380 (3)
C13—H13C	0.9800	C40—H40	0.9500
C14—H14A	0.9800	C41—C42	1.394 (2)
C14—H14B	0.9800	C41—H41	0.9500
C14—H14C	0.9800	C42—C43	1.478 (2)
C15—H15A	0.9900	C44—H44A	0.9800
C15—H15B	0.9900	C44—H44B	0.9800
C16—C20	1.414 (2)	C44—H44C	0.9800
C17—C18	1.380 (3)	C45—C46	1.521 (3)
C17—H17	0.9500	C45—H45A	0.9800
C18—C19	1.380 (2)	C45—H45B	0.9800
C18—H18	0.9500	C45—H45C	0.9800
C19—C20	1.397 (2)	C46—C47	1.515 (3)
C19—H19	0.9500	C46—H46A	0.9900
C20—C21	1.479 (2)	C46—H46B	0.9900
C22—H22A	0.9800	C47—C48	1.519 (3)
C22—H22B	0.9800	C47—H47A	0.9900
C22—H22C	0.9800	C47—H47B	0.9900
S2—C38	1.7680 (16)	C48—C49	1.511 (3)
S2—C37	1.8174 (17)	C48—H48A	0.9900
O4—C23	1.378 (2)	C48—H48B	0.9900
O4—H4	0.830 (9)	C49—C50	1.514 (4)
O5—C43	1.328 (2)	C49—H49A	0.9900
O5—C44	1.458 (2)	C49—H49B	0.9900
O6—C43	1.209 (2)	C50—H50A	0.9800
N2—C39	1.342 (2)	C50—H50B	0.9800
N2—C38	1.343 (2)	C50—H50C	0.9800
C23—C28	1.409 (2)		
			
C16—S1—C15	101.14 (8)	C28—C27—H27	118.8
C1—O1—H1	113.9 (16)	C27—C28—C23	116.71 (14)
C21—O3—C22	114.81 (13)	C27—C28—C29	120.98 (14)
C16—N1—C17	118.41 (15)	C23—C28—C29	122.26 (14)
O1—C1—C2	122.82 (15)	C30—C29—C32	106.08 (16)
O1—C1—C6	114.38 (14)	C30—C29—C28	111.62 (14)
C2—C1—C6	122.80 (15)	C32—C29—C28	109.91 (14)
C3—C2—C1	116.55 (15)	C30—C29—C31	106.05 (15)
C3—C2—C11	120.59 (14)	C32—C29—C31	111.57 (15)
C1—C2—C11	122.83 (14)	C28—C29—C31	111.43 (15)
C4—C3—C2	122.51 (15)	C29—C30—H30A	109.5
C4—C3—H3	118.7	C29—C30—H30B	109.5
C2—C3—H3	118.7	H30A—C30—H30B	109.5
C3—C4—C5	119.02 (15)	C29—C30—H30C	109.5
C3—C4—C15	121.38 (15)	H30A—C30—H30C	109.5
C5—C4—C15	119.59 (15)	H30B—C30—H30C	109.5
C6—C5—C4	121.97 (16)	C29—C31—H31A	109.5
C6—C5—H5	119.0	C29—C31—H31B	109.5
C4—C5—H5	119.0	H31A—C31—H31B	109.5
C5—C6—C1	117.13 (15)	C29—C31—H31C	109.5
C5—C6—C7	121.02 (15)	H31A—C31—H31C	109.5
C1—C6—C7	121.85 (15)	H31B—C31—H31C	109.5
C10—C7—C6	111.80 (14)	C29—C32—H32A	109.5
C10—C7—C9	107.03 (15)	C29—C32—H32B	109.5
C6—C7—C9	110.33 (14)	H32A—C32—H32B	109.5
C10—C7—C8	106.82 (15)	C29—C32—H32C	109.5
C6—C7—C8	110.64 (14)	H32A—C32—H32C	109.5
C9—C7—C8	110.11 (14)	H32B—C32—H32C	109.5
C7—C8—H8A	109.5	C36—C33—C24	111.28 (14)
C7—C8—H8B	109.5	C36—C33—C34	107.41 (17)
H8A—C8—H8B	109.5	C24—C33—C34	110.11 (15)
C7—C8—H8C	109.5	C36—C33—C35	106.51 (16)
H8A—C8—H8C	109.5	C24—C33—C35	111.05 (15)
H8B—C8—H8C	109.5	C34—C33—C35	110.37 (16)
C7—C9—H9A	109.5	C33—C34—H34A	109.5
C7—C9—H9B	109.5	C33—C34—H34B	109.5
H9A—C9—H9B	109.5	H34A—C34—H34B	109.5
C7—C9—H9C	109.5	C33—C34—H34C	109.5
H9A—C9—H9C	109.5	H34A—C34—H34C	109.5
H9B—C9—H9C	109.5	H34B—C34—H34C	109.5
C7—C10—H10A	109.5	C33—C35—H35A	109.5
C7—C10—H10B	109.5	C33—C35—H35B	109.5
H10A—C10—H10B	109.5	H35A—C35—H35B	109.5
C7—C10—H10C	109.5	C33—C35—H35C	109.5
H10A—C10—H10C	109.5	H35A—C35—H35C	109.5
H10B—C10—H10C	109.5	H35B—C35—H35C	109.5
C14—C11—C12	106.75 (14)	C33—C36—H36A	109.5
C14—C11—C2	111.75 (14)	C33—C36—H36B	109.5
C12—C11—C2	110.00 (14)	H36A—C36—H36B	109.5
C14—C11—C13	105.85 (14)	C33—C36—H36C	109.5
C12—C11—C13	110.63 (14)	H36A—C36—H36C	109.5
C2—C11—C13	111.68 (14)	H36B—C36—H36C	109.5
C11—C12—H12A	109.5	C26—C37—S2	106.06 (11)
C11—C12—H12B	109.5	C26—C37—H37A	110.5
H12A—C12—H12B	109.5	S2—C37—H37A	110.5
C11—C12—H12C	109.5	C26—C37—H37B	110.5
H12A—C12—H12C	109.5	S2—C37—H37B	110.5
H12B—C12—H12C	109.5	H37A—C37—H37B	108.7
C11—C13—H13A	109.5	N2—C38—C42	122.44 (14)
C11—C13—H13B	109.5	N2—C38—S2	116.86 (12)
H13A—C13—H13B	109.5	C42—C38—S2	120.71 (13)
C11—C13—H13C	109.5	N2—C39—C40	123.92 (17)
H13A—C13—H13C	109.5	N2—C39—H39	118.0
H13B—C13—H13C	109.5	C40—C39—H39	118.0
C11—C14—H14A	109.5	C41—C40—C39	118.20 (16)
C11—C14—H14B	109.5	C41—C40—H40	120.9
H14A—C14—H14B	109.5	C39—C40—H40	120.9
C11—C14—H14C	109.5	C40—C41—C42	119.75 (16)
H14A—C14—H14C	109.5	C40—C41—H41	120.1
H14B—C14—H14C	109.5	C42—C41—H41	120.1
C4—C15—S1	107.74 (11)	C41—C42—C38	117.91 (16)
C4—C15—H15A	110.2	C41—C42—C43	121.06 (15)
S1—C15—H15A	110.2	C38—C42—C43	121.03 (14)
C4—C15—H15B	110.2	O6—C43—O5	123.38 (16)
S1—C15—H15B	110.2	O6—C43—C42	123.82 (15)
H15A—C15—H15B	108.5	O5—C43—C42	112.79 (14)
N1—C16—C20	121.64 (15)	O5—C44—H44A	109.5
N1—C16—S1	116.34 (12)	O5—C44—H44B	109.5
C20—C16—S1	122.00 (12)	H44A—C44—H44B	109.5
N1—C17—C18	123.98 (16)	O5—C44—H44C	109.5
N1—C17—H17	118.0	H44A—C44—H44C	109.5
C18—C17—H17	118.0	H44B—C44—H44C	109.5
C17—C18—C19	117.90 (16)	C46—C45—H45A	109.5
C17—C18—H18	121.0	C46—C45—H45B	109.5
C19—C18—H18	121.0	H45A—C45—H45B	109.5
C18—C19—C20	119.90 (16)	C46—C45—H45C	109.5
C18—C19—H19	120.1	H45A—C45—H45C	109.5
C20—C19—H19	120.1	H45B—C45—H45C	109.5
C19—C20—C16	118.14 (15)	C47—C46—C45	112.9 (2)
C19—C20—C21	120.18 (15)	C47—C46—H46A	109.0
C16—C20—C21	121.65 (15)	C45—C46—H46A	109.0
O2—C21—O3	123.27 (15)	C47—C46—H46B	109.0
O2—C21—C20	124.68 (16)	C45—C46—H46B	109.0
O3—C21—C20	112.05 (14)	H46A—C46—H46B	107.8
O3—C22—H22A	109.5	C46—C47—C48	113.50 (19)
O3—C22—H22B	109.5	C46—C47—H47A	108.9
H22A—C22—H22B	109.5	C48—C47—H47A	108.9
O3—C22—H22C	109.5	C46—C47—H47B	108.9
H22A—C22—H22C	109.5	C48—C47—H47B	108.9
H22B—C22—H22C	109.5	H47A—C47—H47B	107.7
C38—S2—C37	101.46 (8)	C49—C48—C47	115.1 (2)
C23—O4—H4	112.5 (17)	C49—C48—H48A	108.5
C43—O5—C44	115.01 (14)	C47—C48—H48A	108.5
C39—N2—C38	117.77 (15)	C49—C48—H48B	108.5
O4—C23—C28	121.88 (14)	C47—C48—H48B	108.5
O4—C23—C24	115.35 (14)	H48A—C48—H48B	107.5
C28—C23—C24	122.77 (15)	C48—C49—C50	113.2 (3)
C25—C24—C23	116.85 (15)	C48—C49—H49A	108.9
C25—C24—C33	121.13 (15)	C50—C49—H49A	108.9
C23—C24—C33	122.02 (14)	C48—C49—H49B	108.9
C26—C25—C24	122.25 (15)	C50—C49—H49B	108.9
C26—C25—H25	118.9	H49A—C49—H49B	107.8
C24—C25—H25	118.9	C49—C50—H50A	109.5
C27—C26—C25	118.92 (14)	C49—C50—H50B	109.5
C27—C26—C37	121.34 (14)	H50A—C50—H50B	109.5
C25—C26—C37	119.74 (14)	C49—C50—H50C	109.5
C26—C27—C28	122.49 (15)	H50A—C50—H50C	109.5
C26—C27—H27	118.8	H50B—C50—H50C	109.5
			
O1—C1—C2—C3	178.61 (14)	O4—C23—C24—C33	−0.7 (2)
C6—C1—C2—C3	−1.2 (2)	C28—C23—C24—C33	179.78 (16)
O1—C1—C2—C11	0.8 (2)	C23—C24—C25—C26	0.3 (2)
C6—C1—C2—C11	−179.02 (15)	C33—C24—C25—C26	179.69 (16)
C1—C2—C3—C4	0.0 (2)	C24—C25—C26—C27	0.8 (2)
C11—C2—C3—C4	177.91 (15)	C24—C25—C26—C37	−179.40 (15)
C2—C3—C4—C5	0.7 (2)	C25—C26—C27—C28	−1.3 (2)
C2—C3—C4—C15	−179.29 (14)	C37—C26—C27—C28	178.84 (15)
C3—C4—C5—C6	−0.4 (2)	C26—C27—C28—C23	0.8 (2)
C15—C4—C5—C6	179.64 (14)	C26—C27—C28—C29	−176.89 (15)
C4—C5—C6—C1	−0.7 (2)	O4—C23—C28—C27	−179.21 (16)
C4—C5—C6—C7	179.13 (15)	C24—C23—C28—C27	0.3 (2)
O1—C1—C6—C5	−178.31 (14)	O4—C23—C28—C29	−1.5 (3)
C2—C1—C6—C5	1.5 (2)	C24—C23—C28—C29	177.95 (16)
O1—C1—C6—C7	1.9 (2)	C27—C28—C29—C30	−4.8 (2)
C2—C1—C6—C7	−178.29 (15)	C23—C28—C29—C30	177.59 (16)
C5—C6—C7—C10	−1.5 (2)	C27—C28—C29—C32	112.57 (18)
C1—C6—C7—C10	178.27 (15)	C23—C28—C29—C32	−65.0 (2)
C5—C6—C7—C9	117.45 (17)	C27—C28—C29—C31	−123.21 (18)
C1—C6—C7—C9	−62.7 (2)	C23—C28—C29—C31	59.2 (2)
C5—C6—C7—C8	−120.46 (17)	C25—C24—C33—C36	−1.3 (2)
C1—C6—C7—C8	59.3 (2)	C23—C24—C33—C36	178.15 (17)
C3—C2—C11—C14	5.1 (2)	C25—C24—C33—C34	117.73 (18)
C1—C2—C11—C14	−177.15 (15)	C23—C24—C33—C34	−62.9 (2)
C3—C2—C11—C12	−113.29 (16)	C25—C24—C33—C35	−119.72 (18)
C1—C2—C11—C12	64.5 (2)	C23—C24—C33—C35	59.7 (2)
C3—C2—C11—C13	123.47 (16)	C27—C26—C37—S2	86.45 (17)
C1—C2—C11—C13	−58.8 (2)	C25—C26—C37—S2	−93.38 (16)
C3—C4—C15—S1	99.30 (16)	C38—S2—C37—C26	−179.22 (11)
C5—C4—C15—S1	−80.75 (16)	C39—N2—C38—C42	1.0 (2)
C16—S1—C15—C4	163.90 (12)	C39—N2—C38—S2	−179.48 (12)
C17—N1—C16—C20	0.5 (2)	C37—S2—C38—N2	−6.51 (14)
C17—N1—C16—S1	−178.10 (14)	C37—S2—C38—C42	173.05 (13)
C15—S1—C16—N1	2.45 (15)	C38—N2—C39—C40	−0.9 (3)
C15—S1—C16—C20	−176.15 (14)	N2—C39—C40—C41	0.3 (3)
C16—N1—C17—C18	−1.0 (3)	C39—C40—C41—C42	0.3 (3)
N1—C17—C18—C19	0.2 (3)	C40—C41—C42—C38	−0.2 (2)
C17—C18—C19—C20	1.1 (3)	C40—C41—C42—C43	179.85 (16)
C18—C19—C20—C16	−1.6 (3)	N2—C38—C42—C41	−0.4 (2)
C18—C19—C20—C21	176.41 (16)	S2—C38—C42—C41	−179.96 (12)
N1—C16—C20—C19	0.8 (3)	N2—C38—C42—C43	179.52 (15)
S1—C16—C20—C19	179.29 (13)	S2—C38—C42—C43	0.0 (2)
N1—C16—C20—C21	−177.18 (15)	C44—O5—C43—O6	0.3 (3)
S1—C16—C20—C21	1.3 (2)	C44—O5—C43—C42	−179.87 (17)
C22—O3—C21—O2	3.8 (2)	C41—C42—C43—O6	−168.10 (16)
C22—O3—C21—C20	−175.61 (14)	C38—C42—C43—O6	12.0 (3)
C19—C20—C21—O2	−174.27 (18)	C41—C42—C43—O5	12.1 (2)
C16—C20—C21—O2	3.6 (3)	C38—C42—C43—O5	−167.86 (15)
C19—C20—C21—O3	5.1 (2)	C45—C46—C47—C48	−177.04 (18)
C16—C20—C21—O3	−176.95 (15)	C46—C47—C48—C49	179.34 (18)
O4—C23—C24—C25	178.72 (15)	C47—C48—C49—C50	−179.21 (19)
C28—C23—C24—C25	−0.8 (3)		

### Hydrogen-bond geometry (Å, º)

Cg is the centroid of the C1–C6 ring.

**Table:**

*D*—H···*A*	*D*—H	H···*A*	*D*···*A*	*D*—H···*A*
O1—H1···O6	0.83 (1)	2.22 (2)	2.767 (2)	124 (2)
O4—H4···*Cg*^i^	0.83 (1)	3.25	?	?

Symmetry code: (i) −*x*+1, *y*−1/2, −*z*+1/2.
